# The Palatability of Lopinavir and Ritonavir Delivered by an Innovative Freeze-Dried Fast-Dissolving Tablet Formulation

**DOI:** 10.1155/2018/5908167

**Published:** 2018-01-17

**Authors:** David W. Pittman, Alexandra M. Brantly, Alexandra L. Drobonick, Hannah T. King, Daniel C. Mesta, Caroline G. Richards, Manjari Lal, Manshun Lai

**Affiliations:** ^1^Department of Psychology, Wofford College, 429 North Church Street, Spartanburg, SC 29303, USA; ^2^PATH, 2201 Westlake Avenue, Suite 200, Seattle, WA 98121, USA

## Abstract

Negative hedonic sensory qualities of HIV antiretroviral drugs often reduce patient adherence particularly in pediatric populations requiring oral consumption. This study examines the palatability of an innovative delivery mechanism utilizing a freeze-drying-in-blister approach to create fast-dissolving tablets (FDTs) containing a fixed-dose combination of lopinavir and ritonavir (LPV/r). Consumption patterns of solutions during brief-access and long-term testing and baby foodstuff consumption were analyzed to evaluate the orosensory detection and avoidance of placebo FDTs containing no LPV/r (FDT−) and FDTs containing LPV/r (FDT+). Rats showed no change in consumption patterns for the placebo FDT− compared with control solutions. Rats can detect but do not avoid FDT+ at body-weight-adjusted dosages in both brief-access (30-s) and long-term (23 h) consumption tests. There is an aversive response to concentrated doses of FDT+ during brief-access tests that cannot be masked by 25% sucrose. However, the strongest FDT+ concentration was not rejected when mixed with 50 g of applesauce, banana sauce, or rice cereal baby foodstuffs. The averseness of the FDT+ was associated with the presence of LPV/r and not the FDT− formulation itself. The novel FDT formulation appears to be a palatable delivery mechanism for oral antiretroviral pharmaceuticals especially when mixed with baby foodstuffs.

## 1. Introduction

As of 2015, there were an estimated 1.8 million children under the age of fourteen living with HIV, and only half of those children were receiving medical treatment [1]. One of the most common and successful treatments for HIV is antiretroviral therapy through pharmaceuticals such as the protease inhibitors lopinavir and ritonavir (LPV/r) [2]. LPV/r effectively reduces circulating HIV which in turn has been shown to reduce the risk of opportunistic infections in a pediatric population [3]. Despite a recommended twice-daily dosing regimen of LPV/r for patients as young as 14 days old [4], and even with recently approved LPV/r pellet formulation, poor palatability still exists, posing a challenge in patient compliance [5]. Furthermore, the current oral liquid formulation of LPV/r contains 42% ethanol (EtOH) and 15% propylene glycol which elicits an aversive response. In children under the age of four, taste and the inability to swallow were the most commonly reported problems with the adherence for either liquid or minitablet formulations of LPV/r [6, 7].

The feasibility of an innovative formulation developed using water soluble excipients to effectively deliver combination hydrophobic drugs LPV/r to newborns and infants was recently reported by PATH [8]. Using a freeze-drying-in-blister approach to create a fast-dissolving tablet (FDT) containing LPV/r, PATH was able to reconstitute a single dose of LPV/r with less than 1 mL of water. This small quantity of liquid allows for easier oral consumption of the antiretroviral drugs or the ability to add the drug to a small amount of baby foodstuff for consumption. In addition, the FDTs are heat and humidity stable eliminating the necessity for cold-chain transportation and storage required for the current oral liquid LPV/r medication.

Although ultimately of greatest interest, the use of a human taste panel to screen pharmaceutical drugs for palatability is not cost-effective or feasible in a timely manner. Furthermore, screening drug palatability in a human pediatric population presents even more challenges. The Sprague-Dawley rat is an invaluable model for examining taste perception as there is vast background knowledge of the rodent gustatory system with considerable evidence of similarities to the human gustatory system and taste perceptions [9–14]. The rat behavioral avoidance taste model has been validated as a useful surrogate test to quickly screen compounds that may produce bitter taste and masking agents that may increase the palatability of the drugs [15]. The goal of this study was to assess patterns of consumption related to the palatability of FDTs containing LPV/r when presented in brief-access gustatory palatability tests, long-term solution consumption tests, and foodstuff consumption tests. Given that the LPV/r within the freeze-dried FDTs may still elicit a bitter taste, additional goals of this study sought to identify concentrations of the bitter tastant quinine-HCl that match the licking behavior to the FDTs containing LPV/r and identify potential taste-masking agents to reduce any averseness associated with the drug or comparable quinine concentrations.

## 2. Materials and Methods

### 2.1. Subjects

Adult, male Sprague-Dawley rats, average body weight 264.6 ± 8.4 g (Charles River Laboratories, Raleigh, NC) were individually housed in transparent plastic cages in a temperature-controlled colony room on a 12-12-h light-dark cycle with lights out 30 minutes prior to the start of daily test sessions. Unless otherwise noted, rats had ad libitum access to Teklad 8604 rodent chow and deionized water. All experiments were conducted in accordance with the National Institutes of Health* Guide for the Care and Use of Laboratory Animals*, and all procedures were approved by the Institutional Animal Care and Use Committee of Wofford College.

### 2.2. Chemical Stimuli

All experimental stimuli were mixed daily immediately prior to each test session. In the brief-access gustatory palatability experiment, licking was assessed to four concentrations of LPV/r fast-dissolving tablets (FDT+) prepared as previously described [8]. Each FDT contains 80 mg lopinavir (LPV) and 20 mg ritonavir (r). The concentrations of FDT+ listed in order of increasing concentration were FDT 1 : 30 (1 tablet per 30 mL deionized water); FDT 1 : 3 (1 tablet per 3 mL deionized water), FDT 1 : 2 (1 tablet per 2 mL deionized water), and FDT 1 : 1 (1 tablet per 1 mL deionized water). The dilution of the FDT containing LPV/r at a ratio of 1 tablet to 30 mL solution is a body-weight-adjusted (BW) dosage for the rat (266 g) comparable to a 6-month-old human male at the 50 percentile of growth (8 kg). In addition to the FDT+, a placebo condition of a fast-dissolving tablet containing no LPV/r (FDT−) was also assessed. Three concentrations of the bitter taste stimulus quinine-HCl (1.5, 2.0 and 2.5 mM) were tested alone and with the addition of either 250 mM (8% w/w) or 750 mM (25% w/w) sucrose as taste-masking stimuli. Deionized water was always included as a test stimulus.

In the 23-h fluid consumption experiment, the placebo condition of an FDT− was tested along with the body-weight-adjusted dosage of LPV/r in FDTs (1 FDT+ : 30 mL water ratio) by dissolving 3 FDT+ or 3 FDT− in 3 mL of deionized water and adding 87 mL of either deionized water or whole milk to produce the following stimuli: water alone, water with FDT−, water with FDT+, milk alone, milk with FDT−, and milk with FDT+. The current liquid oral formulation of LPV/r added to 42% EtOH (42% EtOH LPV/r) and a vehicle 42% EtOH alone (42% EtOH) were also tested along with a body-weight-adjusted dosage of 3 mL of 42% EtOH with or without LPV/r added to 87 mL deionized water.

In the foodstuff consumption experiment, one FDT+ or one FDT− was dissolved in 1 mL deionized water and mixed thoroughly into 50 g samples of one of three baby foodstuffs: applesauce (Gerber 2nd Foods, Sitter, Walmart), banana sauce (Gerber 2nd Foods, Sitter, Walmart), or rice cereal single grain (10 g Gerber Rice Cereal, Supported Sitter, Walmart, plus 40 mL whole milk) to produce the following test stimuli: applesauce (AS), AS with FDT−, AS with FDT+, banana sauce (BS), BS with FDT−, BS with FDT+, rice cereal (RC), RC with FDT−, and RC with FDT+.

### 2.3. Brief-Access Gustatory Palatability Tests

Behavioral responses to taste solutions were assessed in an MS-160 contact lickometer (DiLog Instruments, Tallahassee FL). The MS-160 measures rat licking behavior at a resolution of 1 ms during the controlled presentation of up to 16 taste stimuli, as previously described [16, 17]. The MS-160 is housed within an acoustic isolation chamber with 7 dB of white noise. An intake fan and an exhaust fan are mounted on either side of the chamber to produce constant airflow along the axis of the stimulus delivery tray thus diluting and mixing potential olfactory cues during stimulus presentations. Rats (*n* = 13) were placed on a 23-h water restriction schedule during two training days and the duration of testing. Access to 1 h of supplemental water was given 15 minutes after the conclusion of each daily test session. The test stimuli were grouped into four stimulus sets which were presented in a single daily test session in the following order: quinine-HCl concentrations, FDT− and FDT+ concentrations, quinine-HCl plus sucrose-masking solutions, and FDT+ plus sucrose-masking solutions. Each test session consisted of three blocks of randomly ordered test stimuli, including deionized water with stimulus durations of 30 s, a wait time for the first lick of 30 s, and 10-s intervals in between trials.

The total number of licks, latency until first lick, and average interlick interval (ILI) per stimulus were averaged across the three presentations of each stimulus within each daily test session. Consumption of palatable taste solutions results in maximal licking rates with consistently brief ILIs. As taste solutions become less palatable and more aversive the number of licks decreases and the length of ILIs increases. All rats sampled at least one trial of each stimulus during each daily test session. All licking data were converted to a standardized lick ratio [18, 19], which accounts for individual differences in local lick rates independent of motivational state by dividing the licks for each stimulus by the subject's maximum potential lick rate. The maximum potential lick rate was determined by dividing the duration of the stimulus trial by the average ILI (in the range of 125 to 211 ms) during water training tests. This number represents the greatest number of licks each individual rat could perform in a 30-s-duration trial. By dividing the average licks per stimulus by the maximum possible number of licks, the standardized lick ratio becomes a ratio where 1.0 equals the maximum number of licks possible for each individual rat, 0.5 represents half of the maximal number of licks, and 0.0 represents no licks. The latency until the first lick is a measurement of stimulus control and the ability or lack thereof for nonoral cues, such as olfaction, to influence licking behavior. If the rat can detect the stimulus prior to licking the stimulus through either visual or olfactory cues, then the time to approach the spout and initiate the first lick will be longer for that stimulus. Rats had a wait time of 30 s to approach and initiate the trial (30 s duration) with their first lick. Consistent latencies to the first lick that are less than 10 s provide good evidence that rats are motivated to sample each solution with no influence of external cues. An omnibus repeated measures analysis of variance (ANOVA) was performed for all solutions. Post hoc ANOVAs were performed for each set of fluid type with pairwise comparisons (Bonferroni correction) identifying sources of significant ANOVA effects.

### 2.4. 23-h Fluid Consumption Tests

Consumption of solutions during 23-h test sessions was measured by an AC-108 single-spout contact lickometer (DiLog Instruments, Tallahassee, FL). The AC-108 measures licking behavior of up to eight spout lickometers simultaneously at a resolution of 1 ms for up to 23-h periods as previously described [20]. Three days prior to testing, rats (*n* = 8) were trained to lick in the AC-108 using a 23-h water restriction period followed by a 60-minute presentation of 200 mM sucrose in the AC-108. After training, rats were returned to ad libitum water access. Testing was conducted in two replicated phases. Using a within-subject design, each phase consisted of ten consecutive daily 23-h test sessions from 1500 to 1400 the following day. Between each test session from 1400 to 1500 the solutions were changed, chow consumption was measured, rats were weighed, and cages were cleaned. For the first eight test sessions, rats were given solutions in a counterbalanced design (Table 1). Due to the aversive nature of 42% EtOH and 42% EtOH + LPV/r, these stimuli were tested on days 9 and 10. Rats consumed very little of these aversive solutions, essentially inducing a water deprivation state during each 23-h test session. After each of these test sessions rats were given ad libitum water access during the 1-h break in testing from 1400 to 1500 to ensure the rats did not begin the next 23-h test session in a water-restricted state. After completion of test phase 1, rats were returned to their home cages with ad libitum water and food access for 14 days before test phase 2, which replicated phase 1 to provide a total of 16 data points for each condition.

Licks for each rat during the 23-h test sessions were grouped into meals initiated by 5 licks within 1 s and terminated by a pause of 600 s or greater. Consumption behaviors during the 23-h test session were quantified by total licks per session, latency until the first lick of the session, number of meals per session, chow consumption, and change in body weight. The pattern of licking within each meal of the test session was characterized through a microstructure analysis of licking behavior as previously described [20–23]. This analysis identifies whole-meal measures (meal lick count and meal duration) and intrameal licking patterns (number of bursts, size of bursts, mean burst duration, mean pause duration, and average lick rate in licks/s) in order to characterize taste-guided behaviors and behaviors influenced by postingestive feedback. Oromotor coordination was assessed by analysis of interlick intervals (ILIs) and the duration of tongue contact with the fluid spout. Licking bursts were defined by a 1-s pause in licking. Mean burst duration is the average length of time for each licking burst within the meal. Mean pause duration is the average length of time from the termination of a burst to the initiation of the next licking burst. Average lick rate was calculated by dividing the meal licks by the meal duration to determine the average number of licks per second. Contact duration was the duration in which the tongue made contact with the spout sufficient to provide electrical bridging. The number of ILIs in the 250–1,000-ms range was divided by the total number of ILIs from 50 ms to 1,000 ms, to yield an ILI ratio (%) reflecting the number of ILIs in the 250–1,000-ms range relative to the majority of ILIs in the meal. The meal pattern analysis variables were averaged across all meals within a single test session. An omnibus repeated measures ANOVA was performed using solutions and phases 1 and 2 as experimental variables for the 16 test sessions. There were no significant differences between phase 1 and phase 2 for any of the dependent variables; therefore the results were combined and post hoc repeated measures ANOVA was performed for each set of stimulus type with post hoc pairwise comparisons (Bonferroni correction) identifying sources of significant ANOVA effects.

### 2.5. Foodstuff Consumption Tests

All training and testing were conducted in 1 h sessions in standard transparent plastic cages. Foodstuffs (50-g samples) were placed in a heavy circular glass container (10 cm diameter) in the corner of the otherwise empty cage. All test sessions were videotaped in high-definition (1020 × 720) for offline behavioral analysis. Seven days prior to testing, rats (*n* = 9) were exposed to each foodstuff (AS, BS, and RC) in a 1-h training session to reduce neophobic responses during testing. Rats (*n* = 9) were given each foodstuff condition once in a counterbalanced design across nine consecutive days, as described in Table 2.

Feeding behavior was quantified through video analysis by experimenters who were blind to the test condition. Total food consumption (g) was used to calculate total calories consumed. Latency to first consumption, time spent feeding, time spent not feeding, number of feeding bouts (defined by a pause of feeding greater than 1 s), and the length of each feeding bout were measured. An omnibus repeated measures ANOVA was performed for all foodstuffs. Post hoc repeated measures ANOVA was performed for each set of food type with post hoc pairwise comparisons (Bonferroni correction) identifying sources of significant ANOVA effects.

## 3. Results

### 3.1. Brief-Access Gustatory Palatability Tests

**Figure 1:**
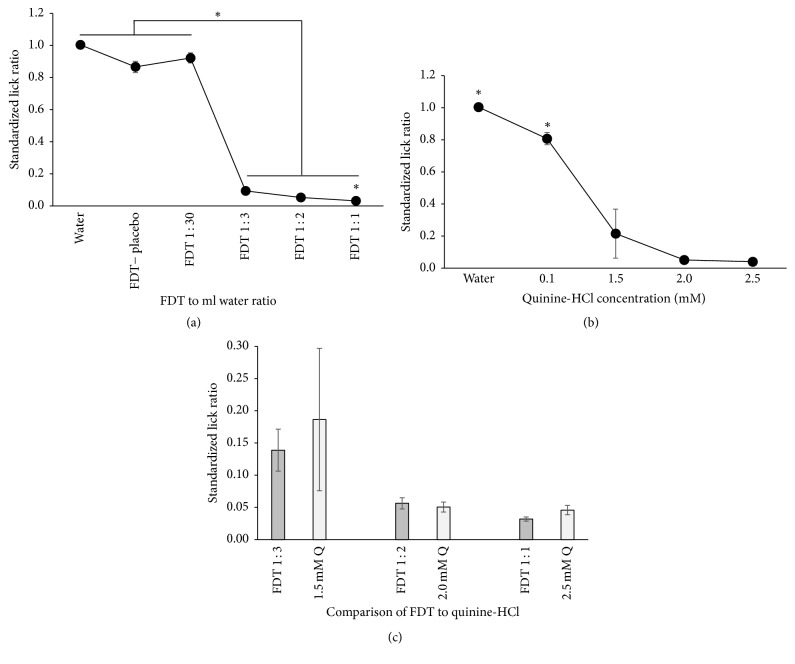
Average licks to stimuli normalized to licks to water in water-restricted rats (*n* = 13) with SEM represented in errors bars. (a) Stars represent significant differences (*p* < .01) in licking to water and body-weight-adjusted FDT+ (FDT 1 : 30) compared with concentrated FDT+ ratios of tablets dissolved in mL water with FDT 1 : 1 having significantly less licks than all stimuli. (b) Stars represent significant differences (*p* < .01) in licking to water and increasing concentrations of quinine-HCl. (c) A comparison of licking to concentrated FDT+ and high concentrations of quinine (Q).

There was a significant main effect (*F*_[5,60]_ = 664.270, *p* < .001) of increasing the LPV/r FDT+ concentration on the standardized lick ratio during 30-s trials (Figure 1(a)) with post hoc pairwise comparisons revealing no difference between water, the FDT− placebo, and the body-weight-adjusted dosage of FDT 1 : 30 ratio (tablet to mL water). All three of these solutions elicited significantly higher licking than the remainder of increased FDT+ concentrations (1 : 3, 1 : 2, 1 : 1; all *p* < .001). Lastly, the strongest concentration of FDT+ (1 : 1) had significantly fewer licks than all of the other stimuli (all *p* < .01). As shown in Figure 1(b), a significant main effect of concentration (*F*_[4,48]_ = 41.288, *p* < .001) revealed that moderate to high concentrations of the prototypical bitter tastant, quinine-HCl, evoked reductions in licking compared with water and a low quinine concentration (0.1 mM).  Figure 1(c) compares the reduced licking for the three highest concentrations of FDT+ to the moderate to high quinine concentrations. Pairwise comparisons reveal no difference in licking between the three matched concentrations of FDT+ and quinine shown in Figure 1(c).

**Figure 2:**
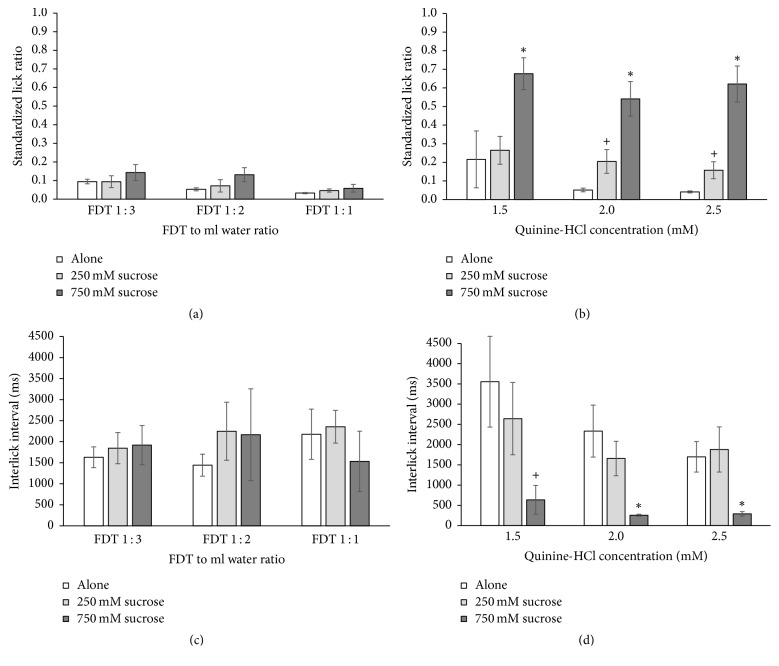
Average licks (a and b) and interlick intervals (c and d) to FDT (a and c) and quinine (b and d) with and without 250 mM or 750 mM sucrose as a masking agent with SEM represented in errors bars. (a) Sucrose did not increase licking to FDT+. (b) Star (*p* < .01) and plus (*p* < .05) symbols represent significant increases in licking when sucrose was added to quinine-HCl. (c) Sucrose did not affect the interlick intervals for FDT+. (d) Star (*p* < .01) and plus (*p* < .05) symbols represent significant decreases in the interlick interval when 750 mM sucrose was added to quinine-HCl.

In an attempt to reduce the averseness of the concentrated FDT+ and quinine, either 250 mM sucrose (8% w/w) or 750 mM sucrose (25% w/w) was added to each concentration of the aversive stimuli. While the addition of sucrose did not significantly increase the licking to FDT+ (Figure 2(a)), there was a main effect of FDT+ concentration (*F*_[2,24]_ = 6.780, *p* = .005) with pairwise comparisons showing FDT 1 : 3 > FDT 1 : 2 > FDT 1 : 1 (all *p* < .01). As shown in Figure 2(b), sucrose was effective at masking the averseness of quinine (*F*_[2,24]_ = 33.491, *p* < .001) with pairwise comparisons showing the addition of 250 mM sucrose significantly increased licking for 2.0 mM and 2.5 mM quinine (both *p* < 0.5) and the addition of 750 mM sucrose increased licking across all three quinine concentrations (all *p* < 0.01) compared with the quinine alone stimuli.

The ILI (duration between each lick in ms) is a measurement of the rate of licking during a trial and can be associated with the palatability of a taste solution. When rats encounter appetitive stimuli, they tend to produce very consistent ILIs in the range of 150–200 ms in duration. When encountering a negative hedonic stimulus, rats will perform stereotypical oromotor responses such as gaping and tongue protrusions in response to the aversive taste stimulus [10]. These gapes and tongue protrusions cause a lengthening of the ILI to the range of 1,000–3,000 ms, as shown in Figures 2(c) and 2(d). In Figure 2(c), it is evident that the two sucrose concentrations did not mask the aversive quality of the FDT+ and there was virtually no change in the long-duration ILIs; however, in Figure 2(d), there is a significant (*F*_[2,24]_ = 9.779, *p* = .001) shortening of the ILI when 750 mM sucrose was added to the quinine concentrations compared with quinine alone (1.5 mM *p* = .047; 2.0 mM *p* = .017; 2.5 mM *p* = .012). This is evidence that 750 mM sucrose effectively suppressed the stereotypical aversive oromotor responses normally elicited by strong concentrations of quinine.

There were no significant effects on the latency to approach and lick the spout for any of the stimuli. Across all trials and stimuli, rats consistently approached and licked the spout within the first 10 s of the trial, demonstrating equivalent motivation to sample the stimuli with no influence of external guiding cues.

### 3.2. 23-h Fluid Consumption Test

**Figure 3:**
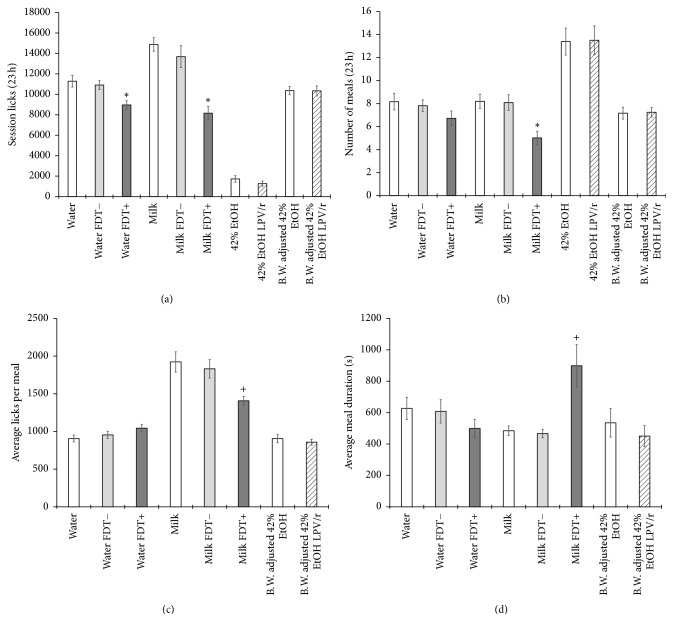
Meal ingestion analysis during 23-h consumption tests with bars representing the means with SEM error bars. Stars represent significant differences (*p* < .01) between solutions containing FDT+ (dark bars) and control solutions (open bars). (a) Total licks to single test solutions. (b) The number of meals defined by pauses greater than 10 minutes. (c) Licks per meal averaged across the test session. (d) Duration of meals averaged across the test session.

**Figure 4:**
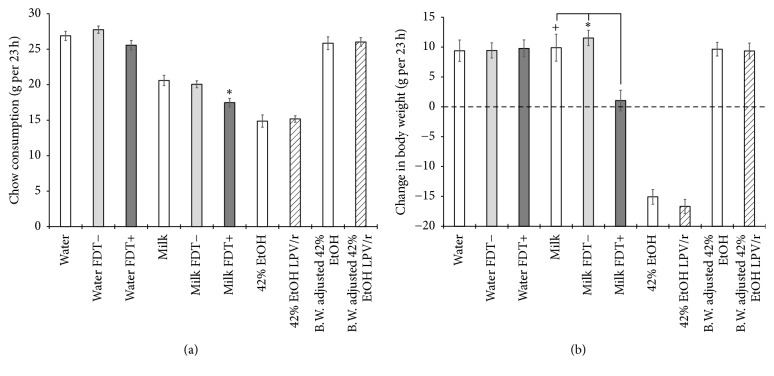
Solid food consumption (a) and changes in body weight (b) during the 23-h solution test sessions. Star (*p* < .01) and plus (*p* < .05) symbols represent significant decreases for the FDT+ compared with control solutions.

There was a slight decrease in the normal consummatory behavior when LPV/r in a fast-dissolving tablet (FDT+ 1 : 30 BW) was added to either water (*F*_[2,28]_ = 10.702, *p* < .001) or milk (*F*_[2,28]_ = 19.395, *p* < .001). As shown in Figure 3(a), the total number of licks in a 23-h test session was slightly lower for both water containing FDT+ and milk containing FDT+ compared with either solution alone or when the placebo FDT− was added to the solutions. Rats showed a strong aversion to both 42% EtOH and 42% EtOH containing LPV/r, failing to consume sufficient fluid in 23 h to meet their daily hydration need. As shown in Figure 4, this water-deprived state significantly reduced chow consumption (*F*_[9,126]_ = 82.090, *p* < .001) and body weight (*F*_[9,126]_ = 47.755, *p* < .001) during the EtOH test sessions (all *p* < .01 compared with all other solutions except each other). There were insufficient licks during the ethanol sessions to allow any meal pattern analysis and therefore the two EtOH solutions were excluded from further meal pattern analyses (Figures 3(c) and 3(d)).

A meal pattern analysis examining the number, duration, and licks per meal during the 23-h test session can identify the source of decreased licking to water with FDT+ and milk with FDT+. While there was a significant decrease in total licks to water with FDT+ compared with the control stimuli, the decrease was so subtle that there were no significant differences in the number of meals, duration of meals, or licks per meal. There was a slight decrease in both the number and duration of meals that likely underlies the overall decrease in total licks to water with FDT−. In contrast, the greater decrease in total licks for milk with FDT+ is reflected in both a significant decrease in the number of meals (Figure 3(b), *F*_(2,28)_ = 9.083, *p* = .001) and the average licks per meal (Figure 3(c), *F*_(2,28)_ = 6.440, *p* = .005). Interestingly there was also a large significant increase in average meal duration (Figure 3(d), *F*_(2,28)_ = 9.835, *p* = .001), indicating a slower rate of ingestion during the meal possibly due to negative postingestive effects produced by milk containing FDT+. Another potential indicator of negative postingestive actions of milk containing FDT+ is a reduction in the ad libitum chow consumed and decreased body weight during the 23-h test session.

Figure 4(a) shows a significant reduction in chow consumption for all of the milk solutions and the two 42% EtOH solutions (*F*_[9,126]_ = 82.090, *p* < .001). The reduction of chow during the EtOH test sessions is likely due to the water/fluid deprivation state while the reduction of chow for milk alone and milk with FDT− is likely due to the extra calories consumed in the milk itself; however, compared with milk alone and milk with FDT−, there was also a further significant decrease in chow consumption, specifically for milk containing FDT+ (*F*_[2,28]_ = 22.666, *p* < .001). The reduction in chow consumption combined with lowering the milk consumption during the test containing FDT+ resulted in a net weight gain of zero over the 23-h test session (Figure 4(b)) compared with a normal gain of 10 g for milk and milk with FDT− (*F*_[2,28]_ = 22.666, *p* < .001).

**Figure 5:**
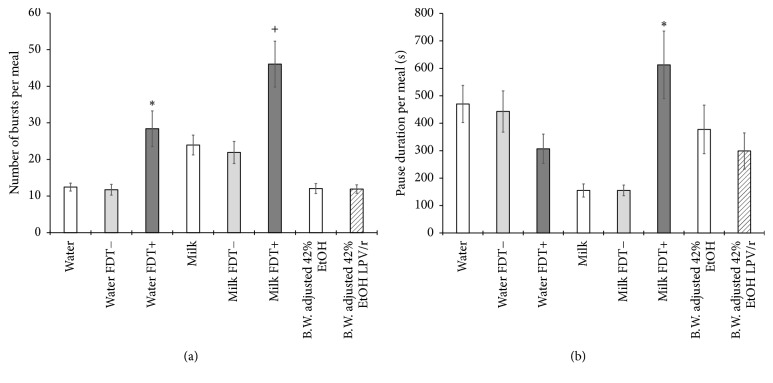
Analyses of licking patterns within meals with bars representing means and SEM error bars. (a) The average number of licking bursts defined by a pause ≥ 1 second. Star (*p* < .01) and plus (*p* < .05) symbols represent significant increases in bursts of licking for FDT+. (b) Cumulative time spent not licking during a meal. The star (*p* < .01) represents a significant increase in time spent paused for FDT+ in milk.

Within each meal, rats consumed the test solutions in a stereotypical manner with bursts of licking separated by pauses greater than 1 s. The number of bursts within a meal significantly increased for both water (*F*_[2,28]_ = 11.363, *p* < .001) and milk (*F*_[2,28]_ = 8.494, *p* = .001) containing FDT+ compared with the control solutions (Figure 5(a)). As each burst is defined by a pause, an increase in the number of bursts directly corresponds to an increase in the number of pauses. The duration of pauses within a meal is related to the palatability of, or motivation to consume, a taste solution. As palatability decreases and the averseness of a taste solution increases the pause durations between licking bursts will increase. The smaller the pause duration is, the more motivated the rat is to consume the solution. As shown in Figure 5(b), both milk alone and milk plus FDT− have very low pause durations, indicating that once rats initiated licking in a meal, their consummatory behavior was very driven, which resulted in fewer and shorter breaks. When FDT+ was added to water, there was no effect on pause duration, indicating that FDT+ alone does not produce a change in the hedonic value of water; however, the addition of FDT+ to milk appears to produce an interaction that results in significantly longer pause durations (*F*_[2,28]_ = 14.798, *p* < .001). This interaction between milk and FDT+ appears to produce a negative hedonic value (demonstrated by more frequent and longer pauses) that decreases normal motivation to consume milk or milk with FDT−.

**Figure 6:**
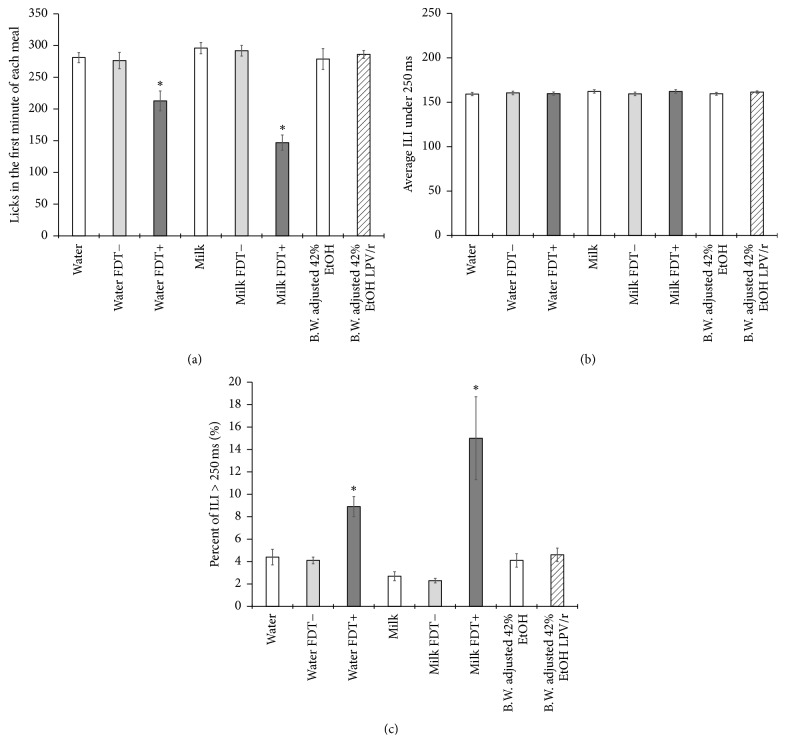
Analyses of intrameal licking patterns associated with palatability with bars representing means and SEM error bars. (a) Licks in the first 60 s of each meal. Stars represent significant (*p* < .01) decreases in licking to the FDT+ solutions. (b) The average interlick interval (ILI) when less than 250 ms. (c) The percentage of interlick intervals in the range of 250–1,000 ms with stars representing significant (*p* < .01) increases in longer ILI for FDT+.

Licking within the first minute of each meal is presumably under the influence of orosensory cues rather than postingestive cues or motivational state.  Figure 6(a) reveals consistent licking at near-maximal rates for all stimuli except water and milk containing FDT+. The addition of FDT+ to water significantly reduced the licks in the first minute of each meal (*F*_[2,28]_ = 12.758, *p* < .001) compared with water alone (*p* = .001) and water plus FDT− (*p* = .004), and the addition of FDT+ to the milk solution also significantly reduced the licks in the first minute of each meal (*F*_[2,28]_ = 70.053, *p* < .001) compared with milk alone (*p* < .001) and milk plus FDT− (*p* < .001). Bursts of licking are the result of a central pattern generator that produces reflexive licking with consistent ILIs between 150 and 170 ms in duration [24–27]. As shown in Figure 6(b), these ILIs have extremely low variability and are consistent across all test stimuli, indicating no impairment or influence on the motor system. Typically less than 5% of the ILIs within a burst exceed 250 ms in duration except when rats encounter an aversive taste stimulus. In that case, rats exhibit stereotypical oromotor behaviors of gaping (wide stretches of the mouth) and lateral tongue protrusions between licks, increasing the percentage of ILIs in the range of 250 to 1,000 ms. As shown in Figure 6(c), the addition of FDT+ to both water (*F*_[2,28]_ = 24.453, *p* < .001) and milk (*F*_[2,28]_ = 11.857, *p* < .001) significantly increases the percentage of ILIs greater than 250 ms compared with water and milk control solutions.

### 3.3. Foodstuffs Consumption

**Figure 7:**
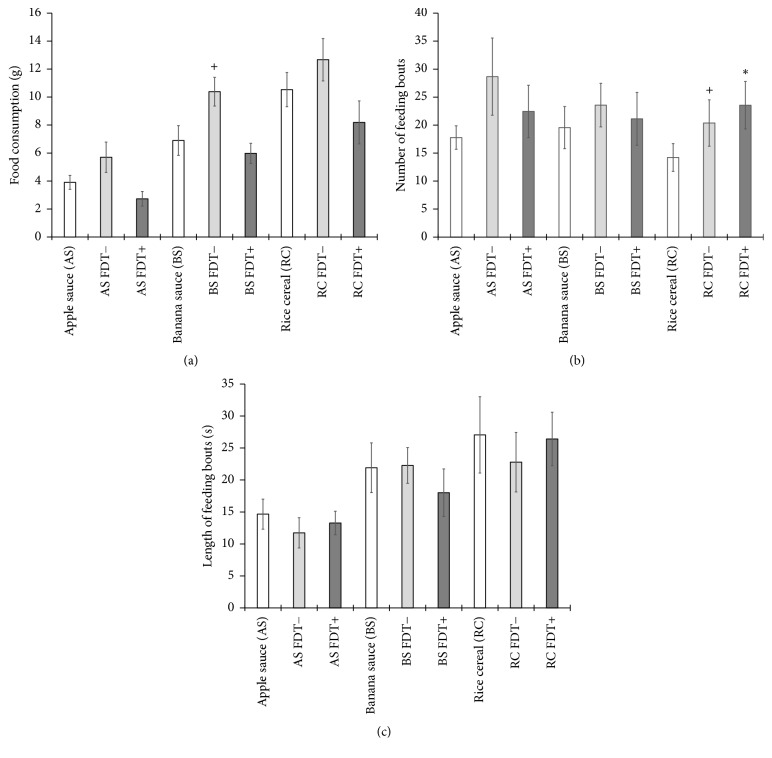
Consumption patterns of applesauce (AS), banana sauce (BS), or rice cereal (RC) alone (open bars), with the FDT− placebo (light bars) and the FDT+ (dark bars) with bars representing means with SEM error bars. Star (*p* < .01) and plus (*p* < .05) symbols represent significant increases compared with control foodstuffs (open bars). (a) Total grams consumed during the 1-h consumption test. (b) Average number of feeding bouts defined by a pause ≥ 1 second. (c) Average length of each feeding bout.

Overall, the consumption of foodstuffs was significantly different (*F*_[2,16]_ = 16.420, *p* < .001), with increasing consumption from AS < BS < RC, which was the most palatable foodstuff.  Figure 7(a) shows the consumption of the three foodstuffs with and without FDT− and FDT+. There was a significant main effect of condition for all three foodstuffs: AS (*F*_[2,16]_ = 8.113, *p* = .004), BS (*F*_[2,16]_ = 7.431, *p* = .005), and RC (*F*_[2,16]_ = 4.377, *p* = .030), indicating that the pattern of food consumption varied across conditions. However, post hoc pairwise comparisons yield no significant differences between the foodstuff conditions for AS or RC, meaning that no comparisons between any two conditions could account for the significant main effect. There were significant pairwise comparisons for the BS, revealing significantly more consumption of BS with FDT− compared with BS alone (*p* = .041) or BS with FDT+ (*p* = .030).

The latency until first consumption can be a measure of palatability with more palatable foods having a shorter latency until first consumption. A significant main effect of foodstuffs (*F*_[2,16]_ = 4.377, *p* = .030) indicated that RC was more palatable with pairwise comparisons, showing that RC had shorter latencies (20.2 s) than both AS (163.0 s, *p* = .017) and BS (105.3 s, *p* = .039). There was no effect of FDT− or FDT+ condition on the latency to consumption within each foodstuff.

Within the meal, feeding bouts were defined by pauses in consumption greater than 1 s. There was a significant effect (*F*_[2,16]_ = 9.014, *p* = .002) to increase the number of feeding bouts when adding FDT− (*p* = .039) or FDT+ (*p* = .002) to RC compared with RC alone (Figure 7(b)). However, there was no effect of adding FDT− or FDT+ on the length of the feeding bouts within each foodstuff (Figure 7(c)). There was a main effect (*F*_[2,16]_ = 7.854, *p* = .004) of foodstuffs on feeding bout length for RC compared with AS (*p* = .10), again indicating a greater palatability and increased motivation to consume the RC foodstuff. There were no significant effects of FDT− or FDT+ on the calories consumed or the total time spent feeding for any of the foodstuffs.

## 4. Discussion

In the present study, avoidance of LPV/r (FDT+) during brief-access tests demonstrated an aversive orosensory component that was attributed to the LPV/r component and not the FDT formulation (FDT−) and furthermore this averseness could not be masked by sucrose. Long-term solution consumption of a body-weight-adjusted LPV/r (FDT+) dosage was mildly aversive in water and interacted with milk to produce a negative hedonic quality but was significantly more palatable than the body-weight-adjusted standard formulation of LPV/r in 42% EtOH. Finally, small quantities of baby food sufficiently masked any negative sensory components of LPV/r (FDT+), suggesting delivery of the drug in food is the most successful method for patient adherence.

### 4.1. Palatability of FDT with and without LPV/r

Licking during brief-access-duration trials is governed by sensory cues from the oral cavity as opposed to postingestive feedback. When motivated to lick by water restriction, rats will decrease from a maximal lick rate when a stimulus elicits sufficient negative hedonic orosensory cues. Rats did not avoid FDT+ at a body-weight-adjusted dosage but showed strong avoidance of concentrated FDT+ doses. Licking to quinine at high concentrations could be matched to the FDT+ doses providing an index of the potential averseness of the FDT+. When rats consume an aversive tastant, such as quinine, they perform reflexive oromotor responses known as taste reactivity [10]. Grill and Norgren [10] classically defined the taste reactivity rejection responses to strong quinine concentrations as a series of gapes and lateral tongue movements that interrupt a rat's stereotypical rhythmic licking behavior. Gape series typically include 2 to 6 gapes of 166 ms in duration with 85–115-ms intergape intervals followed by singular lateral tongue protrusions (85–215 ms), producing a total interruption in the ILI ranging from 600 to 1,800 ms. Rats motivated by water restriction consistently demonstrate ILIs ranging from 150 to 170 ms when licking neutral or appetitive stimuli; however, as shown in Figures 2(c) and 2(d), there is a substantial increase in the ILIs for the strong quinine and concentrated FDT+ ratios. Negative taste reactivity responses (gapes and lateral tongue movements) likely account for the increased ILI durations in both cases. The similarity between the strong quinine and concentrated FDT+ ILIs suggests that the FDT containing the LPV/r elicits aversive orosensory signals not unlike strong bitter tastants, whereas the ILIs for the placebo FDT− (166.3 ± 8.4) and body-weight-adjusted FDT 1 : 30 (201.9 ± 21.4) did not differ from water (150.7 ± 2.3).

During long-term 23-h tests, rats were able to discern a difference between the placebo FDT− and the FDT+ containing LPV/r, demonstrating a slight reduction in licking to the FDT+ compared with control and FDT− solutions; however, this slight decrease is not indicative of a strong aversive sensation, as seen with the dramatic reduction in 42% EtOH with and without LPV/r (Figure 3(a)). As discussed below, the consumption patterns of milk with FDT+ reveal a potential negative postingestive influence on consumption. However, there was no evidence of postingestive influences on the consumption patterns of water containing FDT+. The decreases in licks during the first minute of each meal and increases in the percentage of ILIs greater than 250 ms suggest that FDT+ can be detected in water and that the orosensory perception is mildly aversive (Figure 6). Thus, masking of the FDT+ may be required for maximal patient adherence. Overall, the long-term consumption patterns support the evidence from brief-access tests that body-weight-adjusted FDT+ is detectable but not strongly aversive to rats and that placebo FDT− is treated no differently than control solutions. Therefore, it can be concluded that the aversive sensory signals are associated with the presence of LPV/r and not the formulation of the freeze-dried FDT suspension.

### 4.2. Masking of FDT Containing LPV/r

While sucrose was sufficient to mask the bitterness of high quinine concentrations, the lack of masking by sucrose of the averseness of FDT+ suggests that FDT+ may elicit negative sensory cues other than bitter taste. As shown in Figure 2(d), 750 mM (25% w/w) sucrose effectively eliminated negative taste reactivity behaviors to the strong quinine as represented by ILIs less than 500 ms. This suggests that sucrose had the ability to mask the strong quinine in a manner that eliminated the gaping and lateral tongue movement reflexes which produce longer ILIs. However, the same 750 mM sucrose had no effect on the ILIs for any of the concentrated FDT+ ratios. This suggests that the negative taste reactivity behaviors were still elicited by the FDT+ formulation even with the addition of sucrose (Figure 2(c)).

In contrast to concentrated FDT+ 1 : 1 ratio avoidance when presented in solution form, when the same single FDT+ in one mL of water ratio was mixed into 50 g of baby foodstuffs, there was no evidence of avoidance. In fact, the number of feeding bouts increased for RC when FDT+ was added to it. The presence of calories in foodstuffs and the ability of complex foodstuffs to mask nongustatory orosensory cues such as tactile sensations are two differences between solution and baby foodstuff testing paradigms that could explain the differential avoidance of FDT+. The inability of caloric content in 750 mM sucrose to mask FDT+ suggests that calories alone are not sufficient to overcome the negative hedonic value associated with concentrated FDT+. Therefore, it is likely that the baby foodstuffs were more efficient at masking negatively associated olfactory, textural, lubricious, or astringent trigeminal sensations potentially associated with FDT+.

### 4.3. Interactions between Milk and FDT+

The pattern of consumption of milk containing FDT+ during the 23-h long-term tests suggests negative postingestive influences. The reduction in licking to milk and FDT+ was due to several factors, including a decrease in the number of licking sessions (meals) and a decrease in the average licks per meal. Accompanying the decrease in licks per meal was a significant increase in meal duration rather than a decrease in meal duration, which might be expected. The increase in meal duration coupled with a decrease in licks per meal reflects a slower lick rate with more frequent and lengthy pauses during the meal and fewer licks per burst. This slower pattern of consumption is associated with negative postingestive influences such as gastric distress [28–30]. Dextran, a polymer bulking agent in the FDT, has been reported to interact with casein proteins in milk through electrostatic interaction, causing aggregation leading to destabilization of milk. It is possible that destabilized milk may have altered properties, thereby impacting the sensory response or digestion in rodents [31–33]. The decrease in chow consumption and body weight selectively during the milk with FDT+ test sessions further supports the premise that negative postingestive feedback such as gastric distress was influencing the consummatory behavior of the rats.

## 5. Conclusions

The novel delivery mechanism of LPV/r contained in freeze-dried FDTs has several innovative improvements to the standard liquid formulation, including heat stability, ease of distribution, and ready reconstitution in a small (1 mL) liquid sample of water [8]. This study suggests that the FDT− placebo formulation itself is palatable and does not influence consumption patterns when dissolved in water, milk, or baby foodstuffs. The presence of LPV/r in the FDT+ formulation is mildly aversive at body-weight-adjusted dosage and strongly aversive at concentrated dosages (1 : 3, 1 : 2, and 1 : 1 FDT+ : mL H_2_O). Sucrose was an ineffective masking agent of the concentrated FDT+; however, baby foodstuffs did effectively mask any negative hedonic qualities of the strongest 1 : 1 FDT+ concentration. Therefore, consumption of the FDT+ with baby food may result in maximal patient adherence for antiretroviral therapy.

## Figures and Tables

**Table 1:** Schedule for the 23-h consumption test sessions of water (W) and milk (MS) alone, with placebo FDT− and with FDT+ and body-weight-adjusted (BW) and full-strength 42% ethanol (EtOH) with and without LPV/r.

	Day 1	Day 2	Day 3	Day 4	Day 5	Day 6	Day 7	Day 8	Day 9	Day 10
Rat 1	W	W + FDT+	W + FDT−	MS	MS + FDT−	MS + FDT+	BW 42% EtOH + LPV/r	BW 42% EtOH	42% EtOH + LPV/r	42% EtOH
Rat 2	W + FDT+	W + FDT−	MS	MS + FDT−	MS + FDT+	BW 42% EtOH + LPV/r	BW 42% EtOH	W	42% EtOH	42% EtOH + LPV/r
Rat 3	W + FDT−	MS	MS + FDT−	MS + FDT+	BW 42% EtOH + LPV/r	BW 42% EtOH	W	W + FDT+	42% EtOH + LPV/r	42% EtOH
Rat 4	MS	MS + FDT−	MS + FDT+	BW 42% EtOH + LPV/r	BW 42% EtOH	W	W + FDT+	W + FDT−	42% EtOH	42% EtOH + LPV/r
Rat 5	MS + FDT−	MS + FDT+	BW 42% EtOH + LPV/r	BW 42% EtOH	W	W + FDT+	W + FDT−	MS	42% EtOH + LPV/r	42% EtOH
Rat 6	MS + FDT+	BW 42% EtOH + LPV/r	BW 42% EtOH	W	W + FDT+	W + FDT−	MS	MS + FDT−	42% EtOH	42% EtOH + LPV/r
Rat 7	BW 42% EtOH + LPV/r	BW 42% EtOH	W	W + FDT+	W + FDT−	MS	W + FDT−	MS + FDT+	42% EtOH + LPV/r	42% EtOH
Rat 8	BW 42% EtOH	W	W + FDT+	W + FDT−	MS	MS + FDT−	MS + FDT+	BW 42% EtOH + LPV/r	42% EtOH	42% EtOH + LPV/r

**Table 2:** Schedule for daily 1-h consumption tests of applesauce (AS), banana sauce (BS), and rice cereal (RC), with placebo FDT− and with FDT+.

	Day 1	Day 2	Day 3	Day 4	Day 5	Day 6	Day 7	Day 8	Day 9
Rat 1	AS	AS + FDT+	AS + FDT−	RC	RC + FDT+	RC + FDT−	BS	BS + FDT+	BS + FDT−
Rat 2	BS	BS + FDT+	BS + FDT−	AS	AS + FDT+	AS + FDT−	RC	RC + FDT+	RC + FDT−
Rat 3	RC	RC + FDT+	RC + FDT−	BS	BS + FDT+	BS + FDT−	AS	AS + FDT+	AS + FDT−
Rat 4	AS + FDT+	AS + FDT−	AS	RC + FDT+	RC + FDT−	RC	BS + FDT+	BS + FDT−	BS
Rat 5	BS + FDT+	BS + FDT−	BS	AS + FDT+	AS + FDT−	AS	RC + FDT+	RC + FDT−	RC
Rat 6	RC + FDT+	RC + FDT−	RC	BS + FDT+	BS + FDT−	BS	AS + FDT+	AS + FDT−	AS
Rat 7	AS + FDT−	AS	AS + FDT+	RC + FDT−	RC	RC + FDT+	BS + FDT−	BS	BS + FDT+
Rat 8	BS + FDT−	BS	BS + FDT+	AS + FDT−	AS	AS + FDT+	RC + FDT−	RC	RC + FDT+
Rat 9	RC + FDT−	RC	RC + FDT+	BS + FDT−	BS	BS + FDT+	AS + FDT−	AS	AS + FDT+
